# The Influence of Specific Bioactive Collagen Peptides on Body Composition and Muscle Strength in Middle-Aged, Untrained Men: A Randomized Controlled Trial

**DOI:** 10.3390/ijerph18094837

**Published:** 2021-04-30

**Authors:** Denise Zdzieblik, Patrick Jendricke, Steffen Oesser, Albert Gollhofer, Daniel König

**Affiliations:** 1 Department for Nutrition, Institute for Sports and Sports Science, University of Freiburg, Schwarzwaldstr 175, 79117 Freiburg, Germany; denise.zdzieblik@cri-mail.org (D.Z.); ag@sport.uni-freiburg.de (A.G.); 2 CRI, Collagen Research Institute, Schauenburgerstr 116, 24118 Kiel, Germany; steffen.oesser@cri-mail.org; 3 Centre of Sports Science, Department for Nutrition, Exercise and Health, University of Vienna, Auf der Schmelz 6, 1150 Vienna, Austria; Daniel.Koenig@univie.ac.at; 4 Department for Nutrition, Exercise and Health, Faculty of Life Sciences, University of Vienna, Althanstrasse 14, 1090 Vienna, Austria

**Keywords:** collagen peptides, whey protein, resistance training, DXA, body composition, muscle strength

## Abstract

It has been shown that specific collagen peptides combined with resistance training (RT) improves body composition and muscle strength in elderly sarcopenic men. The main purpose of this RCT study was to investigate the efficacy of the identical specific collagen peptides combined with RT on body composition and muscle strength in middle-aged, untrained men. Furthermore, in the exploratory part of the study, these results were compared with another group that had received whey protein in addition to the RT. Ninety-seven men completed this study and participated in a 12-week RT program. They ingested 15 g of specific collagen peptides (n = 30; CP-G), placebo (n = 31; P-G), or whey protein (n = 36; WP-G) daily. Changes in fat free mass and fat mass were determined by dual-energy X-ray absorptiometry (DXA), and isometric leg strength was measured. All participants had significantly (*p* < 0.01) improved levels in fat free mass (ΔCP-G = 3.42 ± 2.54 kg; ΔP-G = 1.83 ± 2.09 kg; ΔWP-G = 2.27 ± 2.56 kg), fat mass (Δ CP-G = −5.28 ± 3.19 kg; ΔP-G = −3.39 ± 3.13 kg; ΔWP-G = −4.08 ± 2.80 kg) and leg strength (ΔCP-G = 163 ± 189 N; ΔP-G = 100 ± 154 N; ΔWP-G = 120 ± 233 N). The main analysis revealed a statistically significantly higher increase in fat free mass (*p* = 0.010) and decrease in fat mass (*p* = 0.023) in the CP-G compared with the P-G. The exploratory analysis showed no statistically significant differences between WP-G and CP-G or P-G, regarding changes of fat free mass and fat mass. In conclusion, specific collagen peptide supplementation combined with RT was associated with a significantly greater increase in fat free mass and a decrease in fat mass compared with placebo. RT combined with whey protein also had a positive impact on body composition, but the respective effects were more pronounced following the specific collagen peptide administration.

## 1. Introduction

Characteristics of the aging process include a decrease in muscle mass, strength and functionality. In general, muscle strength and function are assumed to decrease from an average age of about 40 [1,2]. This, together with an increase in fat mass, is often accompanied by orthopedic complaints and an impairment of cardiometabolic risk factors such as increased weight, waist circumference and triglyceride levels, reduced HDL-cholesterol or impaired glucose tolerance [3]. The combination of reduced muscle mass with increased fat mass is defined as sarcopenic obesity, and is associated with a higher mortality risk than sarcopenia or adiposity alone [4]. Interventions aimed at increasing muscle mass and reducing fat mass may therefore help prevent the onset or severity of sarcopenia-related diseases.

An imbalance in caloric intake and energy expenditure contributes to an increase in the number of overweight people or those with obesity. Various studies have shown that regular physical activity, in particular resistance exercise, improves body composition and muscle function [5,6]. Current evidence suggests that the addition of protein may support the effect of strength training on muscle protein synthesis [7]. Dietary proteins are thought to stimulate muscle growth at a cellular level mainly through their content of branched-chain amino acids (BCAA), particularly leucine, via the mTOR-pathway [8]. Nonetheless, the beneficial effect of protein intake—especially regarding the optimal type and amount in different populations—on muscle mass following RT is still under discussion. The current recommendation for the promotion of muscle mass is 1–3 g of leucine or 10–12 g of essential amino acids in younger adults [9]. However, collagen peptides contain only a small percentage of leucine (0.4 g/15 g) [10]. In addition, the total amount of essential amino acids is below the recommendations (about 2.5 g/15 g) of the International Society of Sports Nutrition (ISSN). 

Collagen peptides are more resistant to peptidases due to their small molecule size and the high proportion of proline and hydroxyproline. As a result, collagen peptides are rapidly absorbed from the gastrointestinal tract also in peptide form [11,12,13,14]. Moreover, it has been shown that specific collagen peptides could act as signal messengers in anabolic cellular processes in cartilage, tendons and ligaments [15,16,17], which might be the reason for improved pain symptoms and performance in activity-related joint discomforts [18,19,20], tendinopathy [21,22] and chronic ankle instability [23] in physically active adults.

The stimulation of the muscular anabolic pathways may, therefore, also be enhanced by peptides containing non-essential amino acids rather than only by leucine or other BCAA [24,25,26]. The results of a previous study showed that the intake of 15 g of specific collagen peptides significantly increased fat free mass following RT in older men with sarcopenia [10]. There is evidence of age-related differences in the effects of RT on muscle mass and in the utilization of dietary proteins [27,28].

The aim of the present study was, therefore, to examine the effect of supplementation with 15 g of specific collagen peptides in combination with RT on body composition and muscle strength in order to evaluate if the positive results from a sarcopenic population could be confirmed in a younger male population (30–60 years).

Regarding the results of the previous study with specific collagen peptides [10], the main hypothesis of the main analysis was that also in these younger study participants, the supplementation of specific collagen peptides in combination with RT would improve body composition (increase in muscle mass and decrease in fat mass) and muscle strength compared to placebo.

It is generally accepted that whey protein supplementation in combination with RT programs has a beneficial effect on anabolic processes in the skeletal muscle [7]. In contrast, the scientific knowledge about the effect of collagen peptides in muscle metabolism is less investigated, so far. Therefore, in the exploratory part of the study, the results obtained after taking collagen peptide were compared with the data of a study group that received a daily dosage of 15 g whey protein in combination with RT.

## 2. Materials and Methods

### 2.1. Study Design and Participants

The study was designed as a monocentric, prospective, placebo-controlled, double-blinded trial conducted at the University of Freiburg, Germany. In total, 120 overweight men (body mass index = 27.5 to 35, fat mass > 25%) were recruited. To examine a homogenous age group considering the muscle metabolism, the participants were aged between 30 and 60 years [29]. Based on the data of a previous trial in older men with reduced muscle mass and function [10], the sample size was determined by a power calculation using G*Power (University of Düsseldorf, Düssseldorf, Germany). To ensure a sedentary life-style of the study population, participation in the study was not possible if participants had performed regular physical activity for more than 60 min a week during the past year. Unstable weight and eating behavior were also defined as exclusion criteria, since changes in lifestyle may have an impact on the respective outcome. In this study, participants had to follow a 12-week resistance training in combination with the supplementation of collagen peptides, whey protein or silicon dioxide. Contraindications to physical activity in accordance with American College of Sports Medicine (ACSM) guidelines such as cardiovascular, metabolic or renal diseases [30], or contraindications to the intake of the investigational products diagnosed from anamnestic data also led to an exclusion of the screened participants.

Following written informed consent, participants were assigned to the study groups using a web-based random number generator [31]. Although the whey group was not part of the main analysis, this group was included in the randomization with the same number of participants as in the CP-G and P-G to be sure of minimizing the likelihood of differential treatment or outcome assessments by blinding both participants and researchers. Participants were instructed not to change their diet and physical activities apart from the intake of the product under investigation and the one-hour training session three times a week. In addition, they were asked to complete a three-day nutrition protocol, which included two weekdays and one day at the weekend, both before and after the intervention. All subjects were instructed by a nutritionist on how to quantify the ingested foods using household measurements. The test products were not included in the food record. The protocols were analyzed for daily energy and macronutrient intake using EBISPro 7.0 (EBISPro Stuttgart/Hohenheim, Germany).

The participants were asked to record the time of ingestion and any side effects of the supplement or problems relating to the training program. Blood samples to test routine clinical parameters, including creatine kinase and urea, were taken before and after the intervention. Blood sample collection was performed by a licensed physician.

For t0 (pre-test) and t12 (post-test), the participants were asked to arrive at the University of Freiburg at the same time in the morning and were told to consume the same foods and liquids the day prior to both examinations. Screening began with a medical history questionnaire to ensure the inclusion criteria were met and that there were no risk factors involved that might be aggravated by the exercise protocol.

The examination was approved by the Ethics Committee of the University of Freiburg and registered at the German Clinical Trials Register (DRKS00008925).

### 2.2. Efficacy Outcomes

The primary endpoint of this study was to compare differences in fat free mass between the group receiving collagen peptides (CP-G) and the placebo group (P-G). The respective differences in fat free mass were calculated by subtracting the fat free mass (measured in kg) at the end of the study (t12) from the initial value (t0). Comparing the changes in fat mass, body weight and waist circumference between CP-G and P-G were defined as the secondary endpoints.

The body composition evaluation was performed at baseline and again after three months of intervention by DXA measurement (Stratos DR Dual Fan Beam, Degen Medizintechnik, Heppenheim, Germany). DXA and isometric strength testing were conducted by the same trained and qualified investigator. The DXA scan was calibrated in accordance with the manufacturer’s instructions before each measurement by a phantom scan. While wearing the skintight clothing and no detachable metallic objects, lying straight on the table, participants were subjected to a full body DXA scan to assess various body composition characteristics (fat free mass, explicitly lean mass and fat mass). In addition, the participants had to void bladder before the measurement. Alcoholic beverages and intense exercises had to be avoided 48 h prior to examination.

Comparing the changes in the maximum voluntary isometric contraction (N) between CP-G and P-G was also considered as secondary endpoint. The isometric strength testing was determined as the mean value of pressing three times bilaterally using a 90-degree leg press device with an integrated foot force platform (Kistler^®^, Winterthur, Schweiz). In the exploratory part of the study, the effect of whey protein supplementation (WP-G) on changes in fat free mass, fat mass, bodyweight, waist circumference and muscle strength were determined and compared with CP-G and P-G.

### 2.3. Investigational Products

A specific mixture of bioactive collagen peptides (BODYBALANCE^®^, Gelita AG, Germany) were used for this study. The placebo consisted of silicon dioxide, and the whey protein was a whey isolate (Volactive^®^ 90%, Hertfordshire, UK). Table S1 shows the amino acid composition of the collagen peptides and the whey protein.

All of the test products were packed in single sachets containing a daily dose of 15 g. The powders had to be dissolved in 250 mL of water at room temperature and ingested once daily. Participants were instructed to drink the solution within an hour after training. On days without training, the test products had to be ingested at the same time as on training days.

To check compliance, supplements not used were collected from the subjects at the final visit. In addition, the supplementation was documented in the compliance calendar.

### 2.4. Exercise Intervention Program

Sixty minutes of supervised RT was conducted at the University of Freiburg at the same time of day (8 a.m.–8 p.m.). The protocol was based on recommendations for RT in a healthy untrained population to improve muscular strength and hypertrophy [6]. The sessions were performed three times weekly over a period of 12 weeks. Training began with a ten minute cardio-training (50 to 100 W) to warm up. The three-set program included horizontal leg presses (both legs), reverse crunches, lat-pull exercise, sit-ups and chest presses with 1 to 2 min rest periods between sets. The load was adjusted individually to facilitate the proper execution of the required repetitions as follows: week 1–2: fifteen repetitions with 70% of one repetition maximum (RM), week 3–4: twelve repetitions with 75% of 1 RM, week 5–8: ten repetitions with 80% of 1 RM; week 9–12: eight repetitions with 85% of 1 RM. If all sets were performed with the correct technique, the load was increased by 5 to 10%. Researchers noted the participants’ load for each exercise in every RT session.

The data were evaluated on the basis of all participants that completed the trial and complied with the study protocol (per protocol population). A protocol violation was defined as any notable deviation from the study protocol procedures. An effect of the study supplements could be expected if at least 80% of the supplements were taken [32]. Compliance was therefore monitored by the collection of unused samples, and participants were excluded from the analysis if less than 80% of the supplements were taken. Compliance for resistance training was defined as documentation of at least 30 training sessions of the scheduled 36 sessions. This compliance level was chosen in accordance with the current ACSM recommendations on resistance training [6].

### 2.5. Statistical Analysis

All data are presented as mean ± standard deviation (SD) in tables and text and mean ± the 95% confidence interval (95% CI) in figures. SPSS statistics (IBM SPSS Statistics for Windows, Version 23.0. Armonk, NY, USA: IBM Corp.) was used for all statistical analyses. All of the tests in the descriptive analysis were performed as two-sided tests, and the significance level was set at α = 0.05.

Data distribution was examined with a Shapiro–Wilk test. In case of normal distribution, the homogeneity of the baseline values between the study groups was checked by using one-way ANOVA. Otherwise, the Kruskal–Wallis test was used.

For the main analysis, the mean differences in the primary and secondary endpoints obtained from CP-G and P-G were compared using a linear mixed model (LMM) for continuous variables. The factors were treatment (BODYBALANCE^®^ and placebo) and time (pre- and post-intervention levels). For the exploratory part of the study, mean differences of the same parameters obtained from all of the groups were also compared using LMM for continuous variables. The factors were treatment (BODYBALANCE^®^, placebo and whey protein) and time (pre- and post-intervention levels). Post hoc tests were performed for CP-G vs. WP-G and for P-G vs. WP-G. The comparison of CP-G vs. P-G was already performed in the main analysis. The changes in body composition and muscle strength during the intervention period within the groups were analyzed using the paired sample t-test, or the Wilcoxon signed-rank test when the data could not be assumed to be distributed normally. As a magnitude of the difference between groups, the effect sizes were calculated from differences in means between groups at the end of the investigation (Cohen’s d with correction according to Hedges).

## 3. Results

### 3.1. Subjects

**Figure 1:**
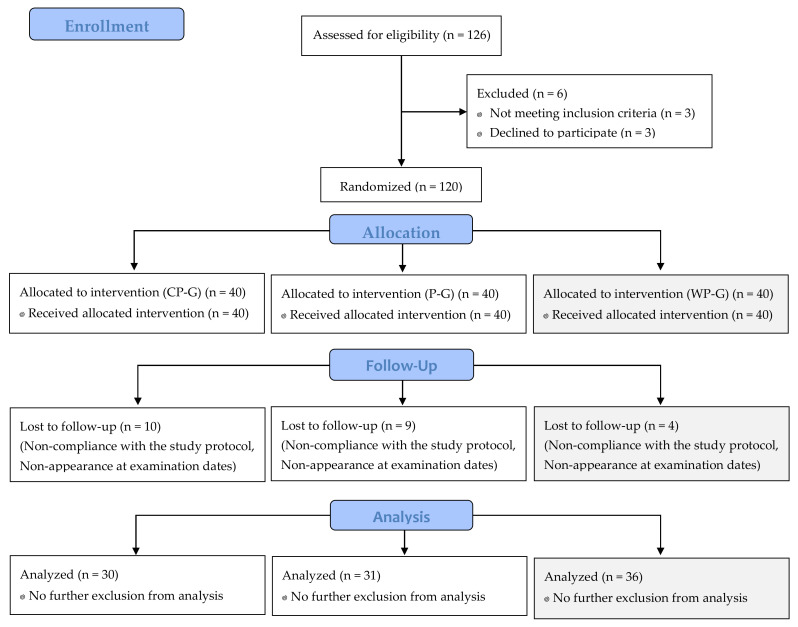
Flow chart of subject recruitment, randomization and follow-up.

A total of 120 adult males met the inclusion criteria and were randomized (Figure 1). The per-protocol population (PP-Population) included 61 participants in the main analysis. The exploratory analysis included 97 participants. Thirty participants in the CP-G, 31 participants in the P-G and 36 participants in the WP-G were analyzed. Reasons for premature study termination or exclusion from the analysis are shown in Figure 1. The exclusion of participants was related to missing the training protocol for >6 times (non-compliance with the study protocol). None of the dropouts were related to side-effects or adverse events caused by the intake of collagen peptides, placebo or whey protein. No adverse events were noted for the PP-population, and no pathological findings were observed in the routine blood tests, including creatine kinase and urea.

The baseline data of the study participants are summarized in Table 1. No statistically significant differences for any demographic result were observed between the study groups of the main and exploratory analysis at the beginning of the study (Table 1).

### 3.2. Main Analysis of Body Composition and Muscle Strength

The baseline data of the respective outcomes of the main analysis are summarized in Table 2. No significant baseline differences between the study groups were detected except for waist circumference. For this parameter, the baseline data were imbalanced with a significantly lower mean waist circumference in the P-G in comparison with the CP-G (*p* = 0.011).

The current investigation identified a significant improvement in fat free mass, fat mass, muscle strength and waist circumference in all groups (Table 2). Moreover, bone mineral content and skeletal muscle mass increased statistically significantly (*p* < 0.05) in all groups during the course of the study.

**Figure 2:**
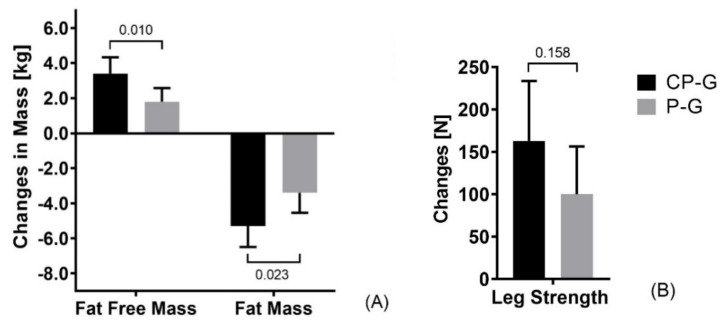
Absolute changes in (**A**) body composition and (**B**) isometric muscle strength at the end of the study compared between groups of the main analysis (CP-G vs. P-G). Data are shown as mean ± 95 % CI; *p* values <0.05 were considered significant.

The main analysis showed that the specific collagen peptide supplementation exhibited a statistically significantly (*p* = 0.010) greater gain in fat free mass (ΔCP-G = 3.42 ± 2.54 kg) than placebo (ΔP-G = 1.83 ± 2.09 kg), as seen in Figure 2. The additional increase in fat free mass by collagen peptide supplementation was also reflected by the medium to large effect size of d = 0.676 compared with placebo. These results are in line with the changes in the estimated skeletal muscle mass (ΔCP-G = 1.23 ± 1.25 kg; ΔP-G = 0.514 ± 1.05 kg; *p* = 0.011; d = 0.621). The decrease in fat mass was also statistically significantly higher (*p* = 0.023) in CP-G (ΔCP-G = −5.28 ± 3.19 kg) compared with P-G (ΔP-G = −3.39 ± 3.13 kg). The clinical relevance of these results was confirmed by the effect size (d = 0.579). Muscle strength increased by 163 N (~16.3 kg) in CP-G and 100 N (~10 kg) in P-G. The higher increase in muscle strength by the additional intake of collagen peptides after the training sessions compared with placebo did not reach the level of significance and had an effect size of d = 0.366. The decrease in waist circumference was more pronounced in the CP-G (−3.41 ± 2.22 cm) compared with the P-G (−2.50 ± 4.19 cm), but the group differences were not statistically significant (*p* = 0.292; d = 0.270).

An intention to treat analysis was also performed. The results of this approach confirmed the findings of the PP-analysis.

### 3.3. Exploratory Analysis of Body Composition and Muscle Strength

As shown in Table 3, the results of the exploratory analysis revealed statistically significant differences between all study groups with respect to fat free mass (*p* = 0.033). No statistically significant differences were observed when comparing the gain in fat free mass in WP-G (ΔWP-G = 2.27 ± 2.56 kg) with the results of the CP-G (*p* = 0.074) or P-G (*p* = 0.442) in the post hoc analysis. In contrast to the collagen peptide supplementation, the whey protein treatment showed a small effect (d = 0.185). Similarly, the changes in estimated skeletal muscle mass in WP-G (ΔWP-G = 0.728 ± 1.27 kg) were not statistically significantly different from the changes in CP-G (*p* = 0.076) or P-G (*p* = 0.460) in the post hoc analysis. Furthermore, the whey protein supplementation had a smaller effect on changes in the skeletal muscle mass (d = 0.184) compared to collagen peptides. Concerning fat mass, no statistically significant difference between all of the groups was observed (*p* = 0.054). The decrease in fat mass in WP-G (ΔWP-G = −4.08 ± 2.80 kg) did not achieve statistical significance equivalent to the differences between CP-G (*p* = 0.111) and P-G (*p* = 0.341) in the post hoc analysis. Again, the intake of whey protein seemed to be less effective than collagen peptides when compared to placebo (d = 0.228).

Participants following RT in combination with whey protein supplementation improved their muscle strength by 120 N (~12 kg). Comparing the changes in muscle strength between WP-G and CP-G (*p* = 0.417) or P-G (*p* = 0.685), a low effect (d = 0.100) and no statistical differences were detected. The decrease in waist circumference in the WP-G (−2.51 ± 4.56 cm) was not statistically significantly different when compared with CP-G (*p* = 0.323) or P-G (*p* = 0.996). Taking the P-G as reference, the additional effect of whey protein supplementation was very small (d = 0.002).

### 3.4. Dietary Intake

There were no significant differences between the participants’ mean energy and macronutrient intakes prior to the first testing session. According to the analysis of dietary behavior (Table 4), the absolute and relative protein intake did not change during the observation period in any of the groups, and none of the participants were protein deficient. During the course of the study, a statistically significant (*p* = 0.043) reduction in energy intake and a tendential decrease in carbohydrate intake (*p* = 0.079) could be observed in the P-G. As a potential consequence, the energy (*p* = 0.016) and carbohydrate intake (*p* = 0.026) in post-intervention assessment differed significantly between CP-G and P-G.

## 4. Discussion

The current investigation observed a significant increase in fat free mass following the 12-week RT program. The increase in fat free mass was significantly higher in the group receiving a daily dosage of 15 g specific collagen peptides compared to placebo. In the exploratory part, whey protein ingestion further improved fat free mass, but this effect was not statistically different from placebo. In addition, there were no significant differences between collagen peptides and whey protein with respect to changes in fat free mass.

To our best knowledge there are no previous studies that have focused on the effects of collagen peptides in a middle-aged, untrained male population. 

Current evidence suggests that an average gain in fat free mass of 1.1 kg can be obtained by a training intervention lasting at least 10 weeks [5]. Current data from controlled trials and meta-analyses suggest that protein ingestion potentiates the effects of RT. In meta-analysis, the mean differences of fat free mass between protein supplementation and placebo is reported as 0.3–1.0 kg [7,34,35]. According to a previous review, gains in lean mass as a result of resistance training combined with protein supplementation in trained and untrained adults ranged from 0.2 to 5 kg [36]. These data are congruent with the findings of the present trial. Taking the placebo as a reference, the effect of whey protein in this trial was an additional increase in fat free mass of 0.5 kg while the administration of the applied specific collagen peptide resulted in a 1.6 kg greater gain in fat free mass.

In a previous investigation, a 12-week RT combined with the supplementation of 15 g of specific collagen peptides showed a positive effect on body composition, which resulted in a 1.3 kg higher gain in fat free mass compared to placebo in elderly sarcopenic men [10]. Newly published data from an investigation with a comparable study designed have shown that the daily intake of 15 g specific collagen peptides combined with a 12-week RT program led to a significantly higher increase in fat free mass and improvement in strength tests compared to the RT program alone [37]. In contrast to the present investigation, the respective study population was young and RT-experienced men. In another study with participants experienced in RT, the effects of collagen peptide intake on reducing muscle fatigue and improving performance were comparable to whey protein [38]. However, the trial did not include a control group without protein supplementation after the training sessions.

Previous studies have shown that changes in muscle mass induced by RT were associated with the mTOR signaling pathway. It has been demonstrated that protein synthesis is controlled by mTOR at numerous levels and that protein supplementation (especially BCAA) will further improve anabolic stimuli in skeletal muscles via the mTOR pathway [8]. However, the positive effects of collagen peptides cannot solely be explained by the amount and composition of amino acids. The amount of leucine and BCAA in collagen does not seem to be sufficient to stimulate the mTOR pathway in a comparable manner than whey protein. On the other hand, previous publications have demonstrated that mTOR is also activated by other amino acids, e.g., glycine [24,26]. Collagen peptides have a high glycine content. Therefore, this amino acid might also be responsible for the stimulatory effect of collagen peptides on mTOR. According to recent results by Kitakaze et al., Hyp-Gly-dipeptides that occur frequently in collagen peptides also stimulate myogenic differentiation via the mTOR signaling pathway. In a recent study with RT experienced male participants, the effect of specific collagen peptides on fat free mass was examined on the gene level using a muscle biopsy. The results of the proteome analysis revealed a higher number of upregulated proteins in the group receiving specific collagen peptides compared to the placebo group. The upregulated proteins in the collagen peptide group were predominantly associated with the metabolism of the contractile elements [39]. Thus, amino acids present in high amounts in collagen peptides could also act as signaling molecules in skeletal muscle anabolism [25]. However, more research is needed to determine the anabolic effects of collagen peptides in the mTOR pathway.

One possible explanation for the lower effects of whey protein on fat free mass is that the amount of whey protein (15 g/d) was insufficient to induce optimal effects. The latest recommendation of the ISSN for high-quality proteins after RT are 20 g (0.25 g/kg body weight) in young and middle-aged adults [9]. By contrast, the sufficient dose of collagen peptides to increase the fat free mass [40] seems to be 10–15 g. The disparity of the results might be related to the different composition and the mode of action of the protein supplements used. It has been widely accepted that whey protein has a stimulatory effect in the muscle metabolism due to their high content in BCAA [8]. In contrast, the effects of collagen peptides cannot be explained by its BCAA content. First experiments suggest that the interaction between the specific collagen peptides and integrin receptors (e.g., subtype α11β1) might be the key factor for stimulation of muscle protein synthesis [41,42].

Other explanations for the higher increase in fat free mass after collagen peptide supplementation include the theory that about 10% of skeletal muscles consist of collagen, which makes a major contribution to the function and biochemical structure of skeletal muscles [43]. In addition, there is a close functional and anatomical connection between muscle and connective tissue [44,45]. Furthermore, the intramuscular ECM regulates the formation, maintenance and differentiation of myosatellite cells facilitating myofiber growth [46,47]. In vitro experiments showed a significant increase in collagen type I and III with collagen peptide treatment, which consequently led to improved tissue stability [15]. This effect was confirmed in a clinical trial that demonstrated an increase in the resilience and strength of connective tissue after collagen peptide intake [48]. Taking the data of the skeletal muscle mass into account, it could be speculated that part of the observed increase in fat free mass might also be related to the enhanced collagen content of the intramuscular connective tissue. However, this assumption needs to be verified by future muscle biopsy and magnetic resonance imaging studies, as the skeletal muscle mass was estimated by a formula according to Kim et al. [33] and not measured directly.

The significant changes in muscle strength can be explained by the RT, which is effective at substantially increasing muscular strength, when performed three times/week [49]. The WP-G and P-G participants achieved almost the same increase in isometric leg strength, whereas in CP-G the gain in muscle strength was more than 1.5 times as high as in P-G (Table 3). A meta-analysis by Peterson et al. reported an average increase in strength of 20–30% irrespective of the measuring method used [5]. These findings correspond to current ACSM data [6]. It must be mentioned, however, that the increase in muscle strength is subject to a large variance [5,6].

The current study furthermore revealed a statistically significant decrease in fat mass in all participants. The LMM provided no statistical levels for changes in fat mass in the overall comparison. However, the LMM analysis revealed that participants who had received collagen peptides decreased their fat mass statistically significantly by 5.3 kg compared with the placebo group with a fat mass reduction of 3.4 kg. This finding is consistent with the results of a previous study on sarcopenic men who received the same dose of the identical collagen peptide composition [10]. In this trial, collagen peptide supplementation led to a statistically significant fat mass reduction of 5.5 kg and a decrease of fat mass by 2.9 kg in the placebo group. The exploratory analysis revealed no statistical differences between WP-G and CP-G or P-G. The divergence of fat mass in this trial might be partly explained by a higher resting energy expenditure caused by the more pronounced gain in fat free mass in the CP-G. This explanatory approach is speculative. Future investigations concerning body composition need to include measurement of the resting metabolism. In the present study, a moderate reduction of 2.3% in the waist circumference could be observed in the WP-G and P-G. In the group of participants that had received the specific collagen peptides the effect was slightly higher with a decrease of 3.1%. It is assumed that considerably enhanced body fat mass—especially visceral fat—is positively correlated with an increased waist circumference and the main cause of the metabolic syndrome [50]. According to a large cohort study, RT is effective in reducing waist circumference due to favorable changes in body composition [51]. These findings are in accordance with newly published data that shows various RT regimes lead to a significant decrease in fat mass and consequently an improved metabolic profile, including the waist circumference in untrained people [49,52]. An additional effect on the waist circumference in the CP-G could result from the intake of the specific collagen peptides due to a greater gain in fat free mass and loss in fat mass.

The results of the bone mineral content suggest that the osteoprotective effect of the intervention is the consequence of the training program [53]. The effects of exercise without or in combination with protein supplementation on bone mass in non-osteoporotic middle-aged men are rather small. There is evidence that resistance training three times per week for four months resulted in a 2–4% gain in bone mineral density (~60–120 g bone mass) [54], which is in line with the current finding.

It is not assumed that the present results can be attributed to the caloric add-on of 60 kcal (251 kJ) of collagen peptides or whey protein. According to the findings of Hall et al., the additional daily energy intake needs to amount to about 215 kcal (900 kJ). A daily intake of a further 40 kcal (170 kJ) would result in a weight gain of 1.8 kg (4 lb) in five years [55]. Furthermore, none of the study groups significantly altered their protein intake or were deficient in protein.

It must be mentioned that the P-G showed a lower energy intake after 12 weeks. The significant differences of energy intake between the CP-G and P-G were associated with differences in the intake of carbohydrates. It seems plausible that there was an underreporting, since the reported energy intake of 2400 to 2800 kcal is equivalent to the energy requirements of normal weight men of the respective age group [56,57]. However, the participants of the current investigation were overweight men. This assumption is supported by the changes in fat mass. Despite a lower energy intake, the fat loss in the P-G was smaller than in the CP-G or WP-G.

This trial has some limitations. With respect to the comparison in the exploratory analysis, there was an unequal sample size (n_CP-G_ = 30; n_P-G_ = 31; n_WP-G_ = 36), but this had no influence on the statistical outcomes. Limitations of the nutritional protocol might entail an overreporting or rather underreporting of foods consumed [58]. As mentioned above, the amounts of whey protein may have been insufficient, but the study was designed so that the amount of whey protein did not differ from the dosage of collagen peptides. It must be mentioned that the demonstrated efficacy is referred to the specific collagen peptides used, since various collagen peptides differ in their composition and hence their bioavailability and mode of action.

Future research should focus on the role of collagen peptides in the direct regulation of anabolic or catabolic processes in skeletal muscles or fat tissue on a cellular level. Possible pathways include, e.g., the stimulation of collagen synthesis and the mTOR pathway.

Moreover, the effect of various collagen peptides that differ in their biochemical properties (e.g., the amino acid sequence) in combination with other training settings needs to be further elucidated.

## 5. Conclusions

This study examined in the main analysis whether the supplementation of specific bioactive collagen peptides in combination with RT three times a week for 60 min induced a statistically significantly higher increase in fat free mass and a greater reduction in fat mass than RT alone. The respective effects were also compared with a group receiving whey protein in an exploratory approach. It was found that, compared to placebo, the ingestion of specific collagen peptides (BODYBALANCE^®^) resulted in a statistically significantly higher gain in fat free mass and loss in fat mass. It was also shown that this greater effect on muscle protein synthesis and reduction in fat mass was less pronounced after whey protein supplementation. However, there was no statistically significant difference in a direct comparison between the two protein supplements. Muscle strength increased significantly in all groups as a result of RT. The respective results refer to the specific collagen peptides used. How these effects are applicable to other collagen peptides needs to be clarified.

## Figures and Tables

**Table 1:** Baseline data (T0) for the analyzed population (n = 97).

	CP-G (n = 30)	P-G (n = 31)	WP-G (n = 36)	*p* Value
Age (y)	51.8 ± 4.56	47.4 ± 7.26	49.6 ± 7.98	0.058 ^#^
Height (m)	1.80 ± 0.07	1.79 ± 0.05	1.80 ± 0.06	0.596 *
Body weight (kg)	99.6 ± 9.00	95.5 ± 10.8	98.3 ± 11.5	0.293 ^#^
BMI (kg/m^2^)	31.0 ± 2.93	29.9 ± 2.56	30.2 ± 2.60	0.416 ^#^
BP sys (mm Hg)	143.3 ± 16.9	136.3 ± 16.0	141.6 ± 16.3	0.241 ^#^
BP dia (mm Hg)	91.5 ± 8.63	87.4 ± 10.3	89.4 ± 9.32	0.180 ^#^

Data represent mean ± SD; differences between all groups tested with * analysis of variance, ^#^ Kruskal–Wallis Test.

**Table 2:** Efficacy outcomes at baseline and following supplementation with collagen peptides or placebo.

	CP-G (n = 30)		P-G (n = 31)		*p* Value LMM
	T0	T12	T0	T12	
Fat free mass (kg)	60.4 ± 4.78	63.8 ± 6.03 ***	58.5 ± 6.29	60.3 ± 5.71 ***	**0.010**
Fat free mass (%)	60.8 ± 4.50	65.3 ± 4.76 ***	61.4 ± 4.31	64.4 ± 5.02 ***	0.023
Skeletal muscle mass ^1^	30.8 ± 2.42	32.1 ± 3.02 ***	30.0 ± 3.18	30.5 ± 2.85 *	0.011
Fat mass (kg)	35.9 ± 7.11	30.6 ± 6.48 ***	33.7 ± 6.89	30.3 ± 7.57 ***	0.023
Fat mass (%)	35.8 ± 4.71	31.1 ± 5.06 ***	35.1 ± 4.53	31.9 ± 5.30 ***	0.031
Bone mineral content (kg)	3.34 ± 0.339	3.57 ± 0.460 **	3.34 ± 0.380	3.49 ± 0.442 *	0.330
Bone mineral content (%)	3.37 ± 0.376	3.66 ± 0.439 ***	3.52 ± 0.371	3.76 ± 0.533 **	0.602
Body weight (kg)	99.6 ± 9.00	98.0 ± 8.49 ***	95.5 ± 10.8	94.1 ± 10.8 *	0.743
Waist circumference (cm)	107.5 ± 6.67 ^†^	104.1 ± 6.49 ***	102.9 ± 7.00 ^†^	100.4 ± 7.77 **	0.292
Muscle strength (N)	1695 ± 378.7	1858 ± 399.2 ***	1706 ± 351.8	1806 ± 365.9 **	0.158

Data represent mean ± SD; *p* value LMM, significance between groups in linear mixed model testing assessing treatment × time interaction; ^†^ = *p* < 0.05 between groups at baseline; * = *p* < 0.05; ** = *p* < 0.01; *** = *p* < 0.001 within the group from baseline to final examination. ^1^ Estimated skeletal muscle mass according to Kim et al. [33]. Bold numbers represent statistical significance of the primary endpoint.

**Table 3:** Efficacy outcomes at baseline and following supplementation with collagen peptides, whey protein or placebo.

	CP-G (n = 30)		P-G (n = 31)		WP-G (n = 36)		*p* Value LMM
	T0	T12	T0	T12	T0	T12	
Fat free mass (kg)	60.4 ± 4.78	63.8 ± 6.03 ***	58.5 ± 6.29	60.3 ± 5.71 ***	59.6 ± 6.22	61.9 ± 6.76 ***	**0.033**
Fat free mass (%)	60.8 ± 4.50	65.3 ± 4.76 ***	61.4 ± 4.31	64.4 ± 5.02 ***	60.8 ± 3.29	64.3 ± 4.67 ***	0.066
Skeletal muscle mass ^1^	30.8 ± 2.42	32.1 ± 3.02 ***	30.0 ± 3.18	30.5 ± 2.85 *	30.5 ± 3.13	31.2 ± 3.33 **	0.037
Fat mass (kg)	35.9 ± 7.11	30.6 ± 6.48 ***	33.7 ± 6.89	30.3 ± 7.57 ***	35.4 ± 6.44	31.3 ± 7.40 ***	0.054
Fat mass (%)	35.8 ± 4.71	31.1 ± 5.06 ***	35.1 ± 4.53	31.9 ± 5.30 ***	35.8 ± 3.45	32.1 ± 4.94 ***	0.083
Bone mineral content (kg)	3.34 ± 0.339	3.57 ± 0.460 **	3.34 ± 0.380	3.49 ± 0.442 *	3.25 ± 0.350	3.40 ± 0.311 **	0.325
Bone mineral content (%)	3.37 ± 0.376	3.66 ± 0.439 ***	3.52 ± 0.371	3.76 ± 0.533 **	3.37 ± 0.393	3.58 ± 0.423 ***	0.642
Body weight (kg)	99.6 ± 9.00	98.0 ± 8.49 ***	95.5 ± 10.8	94.1 ± 10.8 *	98.3 ± 11.5	96.6 ± 11.5 ***	0.899
Waist circumference (cm)	107.5 ± 6.67 ^†^	104.1 ± 6.49 ***	102.9 ± 7.00 ^†^	100.4 ± 7.77 **	105.6 ± 7.11	103.2 ± 7.86 ***	0.561
Muscle strength (N)	1695 ± 378.7	1858 ± 399.2 ***	1706 ± 351.8	1806 ± 365.9 **	1633 ± 370.5	1753 ± 355.7 **	0.444

Data represent mean ± SD; *p* value LMM, significance between groups in linear mixed model testing assessing treatment × time interaction; ^†^ = *p* < 0.05 between groups at baseline; * = *p* < 0.05; ** = *p* < 0.01; *** = *p* < 0.001 within the group from baseline to final examination. ^1^ Estimated skeletal muscle mass according to Kim et al. [33]. Bold numbers represent statistical significance of the primary endpoint.

**Table 4:** Dietary patterns at baseline and following supplementation with collagen peptides, whey protein or placebo.

	CP-G (n = 30)		P-G (n = 31)		WP-G (n = 31)		*p* Value LMM
	T0	T12	T0	T12	T0	T12	
Energy (kcal)	2739 ± 672.8	2863 ± 686.4	2573 ± 585.7	2419 ± 614.9 *	2780 ± 897.6	2619 ± 791.5	0.059
Protein (g)	105.0 ± 20.7	107.0 ± 28.1	102.8 ± 29.1	96.6 ± 28.1	108.1 ± 34.0	103.2 ± 34.2	0.508
Protein (g/kg BW)	1.05 ± 0.209	1.10 ± 0.289	1.09 ± 0.318	1.03 ± 0.304	1.11 ± 0.385	1.08 ± 0.360	0.750
Protein (%)	16.1 ± 2.78	15.6 ± 3.08	16.4 ± 3.45	16.5 ± 3.11	16.2 ± 3.42	16.2 ± 2.65	0.469
Fat (g)	113.3 ± 37.4	116.9 ± 36.4	100.4 ± 24.4	99.6 ± 31.6	116.6 ± 48.2	118.5 ± 48.1	0.855
Fat (%)	37.0 ± 7.37	36.1 ± 5.23	34.2 ± 6.96	36.6 ± 5.12	36.5 ± 5.17	38.6 ± 6.67	0.155
Carbohydrates (g)	282.8 ± 95.6	301.4 ± 85.8	276.8 ± 79.6	253.7 ± 76.6	268.2 ± 92.2	253.0 ± 75.5	0.060
Carbohydrates (%)	42.5 ± 7.91	43.0 ± 6.97	43.8 ± 6.92	43.0 ± 7.11	41.4 ± 7.15	40.1 ± 7.06	0.669

Data represent mean ± SD; *p* value LMM, significance between groups of in linear mixed model testing assessing treatment × time interaction. * = *p* < 0.05 within the group from baseline to final examination.

## Data Availability

Data sharing not applicable.

## Supplementary Materials

The following are available online at https://www.mdpi.com/article/10.3390/ijerph18094837/s1, Table S1: Amino Acid Profile (% of Protein) of the collagen peptides and whey protein.
