# Supplementary material for: The Influence of Specific Bioactive Collagen Peptides on Body Composition and Muscle Strength in Middle-Aged, Untrained Men: A Randomized Controlled Trial
Source: Int J Environ Res Public Health. 2021 Apr 30;18(9):4837. doi: 10.3390/ijerph18094837 (PMC8125453; doi:10.3390/ijerph18094837)
Supplement: Supplementary file 1 [file ijerph-18-04837-s001.zip › ijerph-1201634-supplementary.pdf]

Supplemental Material:

Table S1: Amino Acid Profile (% of Protein) of the collagen peptides and whey protein

Amino Acid	% of Whey Protein	% of Collagen Peptide
**Alanine**	5.0	8.5
**Arginine**	2.1	7.8
**Aspartic Acid**	11.0	5.8
**Cystine**	2.2	0.0
**Glutamic Acid**	18.1	10.1
**Glycine**	1.4	22.1
**Histidine**	1.7	1.2
**Hydrolxylysin**	N/A	1.7
**Hydroxyprolin**	N/A	11.3
**Isoleucine**	6.4	1.3
**Leucine**	10.6	2.7
**Lysine**	9.6	3.8
**Methionine**	2.2	0.9
**Phenylalanine**	3.0	2.1
**Proline**	5.5	12.3
**Serine**	4.6	3.2
**Threonine**	6.7	1.8
**Tryptophan**	1.4	0.0
**Tyrosine**	2.6	0.9
**Valine**	5.9	2.4
